# Validation of a new box trainer-related tracking device: the TrEndo

**DOI:** 10.1007/s00464-012-2187-6

**Published:** 2012-02-21

**Authors:** Pieter J. van Empel, Lennart B. van Rijssen, Joris P. Commandeur, Mathilde G. E. Verdam, Judith A. Huirne, Fedde Scheele, H. Jaap Bonjer, W. Jeroen Meijerink

**Affiliations:** 1 Department of Surgery, VU University Medical Centre, P.O. Box 7057, 1007 MB Amsterdam, The Netherlands; 2 Department of Obstetrics and Gynaecology, Sint Lucas Andreas Hospital, Amsterdam, The Netherlands; 3 Department of Obstetrics and Gynaecology, VU University Medical Centre, Amsterdam, The Netherlands; 4 Department of Psychology, University of Amsterdam, Amsterdam, The Netherlands

**Keywords:** Box trainer, TrEndo, Laparoscopy, Training, Psychomotor skills, Objective assessment, Simulation, Motion tracking, Validity

## Abstract

**Background:**

There is an increasing demand for structured objective ex vivo training and assessment of laparoscopic psychomotor skills prior to implementation of these skills in practice. The aim of this study was to establish the internal validity of the TrEndo, a motion-tracking device, for implementation on a laparoscopic box trainer.

**Methods:**

Face validity and content validity were addressed through a structured questionnaire. To assess construct validity, participants were divided into an expert group and a novice group and performed two basic laparoscopic tasks. The TrEndo recorded five motion analysis parameters (MAPs) and time.

**Results:**

Participants demonstrated a high regard for face and content validity. All recorded MAPs differed significantly between experts and novices after performing a square knot. Overall, the TrEndo correctly assigned group membership in 84.7 and 95.7% of cases based on two laparoscopic tasks.

**Conclusion:**

Face, content, and construct validities of the TrEndo were established. The TrEndo holds real potential as a (home) training device.

Minimally invasive surgery (MIS) has been recognized as a contribution to the field of surgery by the majority of general surgeons, gynecologists, and urologists due to a distinct set of advantages, including lower short-term morbidity and mortality rates for laparoscopic resections compared to open surgery, cosmetic advantages, and an associated improved postoperative recovery [1, 2]. Technical–surgical demands in MIS differ from those in open surgery, including reduced depth perception [3–6], longer instruments, counterintuitive instrument movement, and loss of joint dexterity [7, 8]. The operating room (OR) as a primary, complex, and expensive teaching environment is no longer desirable and it also carries legal and ethical concerns, amplified by increasing pressure on OR efficiency [9–12]. There is a demand for structured objective ex vivo training and assessment of laparoscopic skills prior to implementation in practice.

Simulation-based practice does not put patient safety at risk and avoids interference with the efficiency of health-care resources [13, 14]. Other advantages include practice of (exclusively difficult aspects of) procedures at one’s own pace and with constructive feedback. Therefore, skill acquisition is more efficient. Assessment and therefore a minimal competency level of skills prior to implementation in practice are possible. Non-patient-bound simulation-based practice has already been demonstrated to improve MIS skills [15, 16], which are subsequently transferable to the OR [15, 17–20]. Consequently, many surgical programs are incorporating simulation-based practice into their curriculum. However, evaluation of laparoscopic skills is currently still performed mainly by subjective expert observation [21–24].

The laparoscopic box trainer, a traditional MIS simulator, has been shown to improve MIS skills and appears to be effective as a high-fidelity training device [25–29]. The objective of this study was to investigate face, content, and construct validities of a new motion-tracking device, the TrEndo (Training in Endoscopy, Delft University of Technology, Delft, The Netherlands), implemented on a traditional laparoscopic box trainer. The TrEndo is an augmented-reality (AR) simulator that records various task-efficiency parameters (motion analysis parameters, MAPs) during simulated laparoscopic tasks. Inclusion of a motion-tracking device on the laparoscopic box trainer is a relatively new training option.

Before a surgical simulator can be used as a training and assessment device, its validity should be proven by vigorous and objective evaluation [30, 31]. *Face validity* assesses simulator realism [31–33]. *Content validity* describes the simulator’s usefulness as a training tool [31, 32]. *Construct validity* reflects a simulator’s abilities to discriminate between different levels of competence, e.g., experienced surgeons and novices [33–35].

## Materials and methods

A prospective observational cohort study was conducted in The Netherlands and Belgium between February 1 and November 31, 2010.

### Participants

Participants were divided into two groups based on prior laparoscopic experience. Experts were defined as having performed over 100 basic laparoscopic procedures and were recruited at two Dutch conferences. Medical students with no laparoscopic experience were defined as novices and were recruited at the VU University Medical Center. Trainees in urology, gynecology, and surgery participating in a laparoscopic suturing course organized by the VU University Medical Center at hospitals in The Netherlands and Belgium were additionally recruited for face and content validity evaluation purposes [36]. All participants voluntarily participated in this study. Participants with prior TrEndo experience were excluded. A brief introduction to the TrEndo was given to all participants.

### Systems and hardware

Laparoscopic training boxes (Camtronics Nederland B.V., Son, The Netherlands) simulate an abdominal cavity using an aluminum frame and allow regular insertion of traditional trocars with conventional laparoscopic instruments (B. Braun Medical B.V., Melsungen, Germany) and a camera connected to a video monitor on which the simulated environment is viewed.

**Fig. 1:**
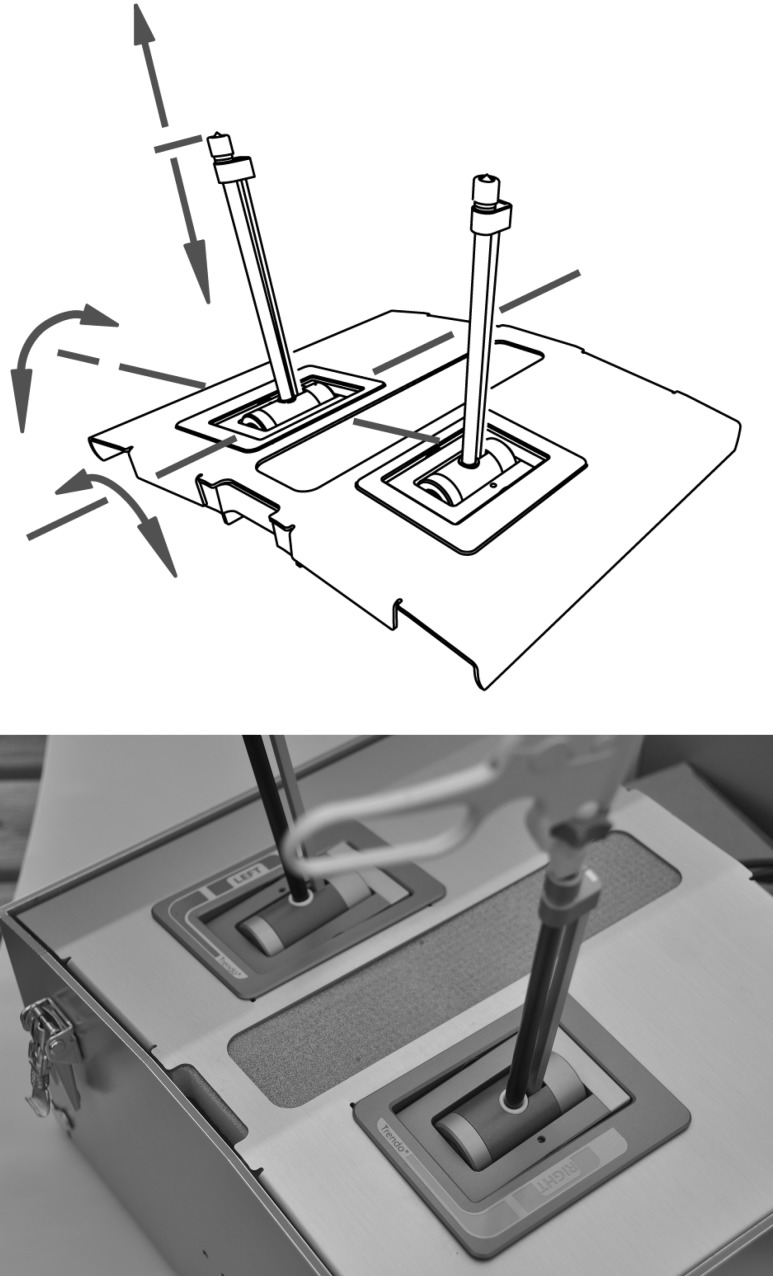

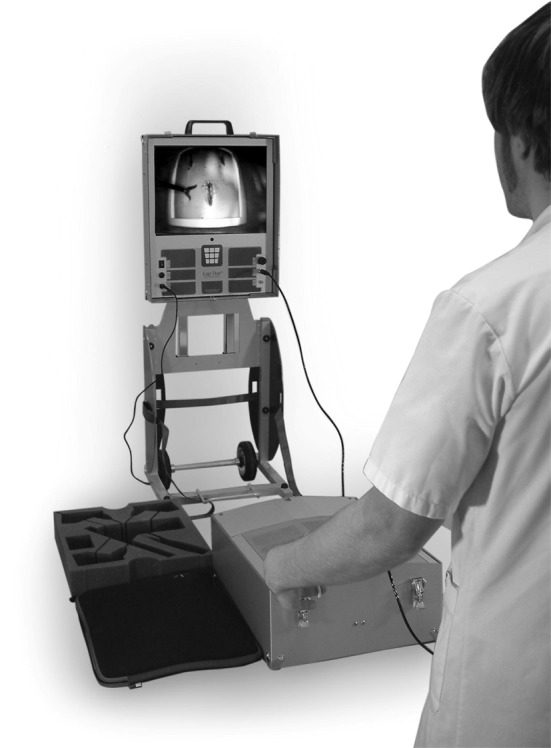
**A** Schematic and photo of TrEndo motion-tracking device on a laparoscopic box trainer. **B** Knot-tying task on a laparoscopic box trainer equipped with the TrEndo tracking device

The TrEndo is constructed as a trocar on a laparoscopic training box through which laparoscopic instruments may still be regularly inserted (Fig. 1). Instrument movement is measured in four degrees of freedom: *X*, *Y*, and *Z* axes and axis rotation [37]. Five motion analysis parameters (MAPs) are recorded with a sample frequency of 100 Hz individually for the right and left hands, including: path length (mm, length of curve described by the tip of the instrument), insertion distance (mm, total distance traveled by the instrument along its axis), angular area (deg^2^, related to the distances between the farthest positions of the instrument during a task), volume (mm^3^, thee-dimensional space used), and time (s) [38].

### Face and content validities

Twenty questions adapted from a previously described study on the face validation of another laparoscopic training device were used to inquire experts’ and trainees’ first impression of the TrEndo with a laparoscopic box trainer [33]. Participants additionally rated six possible advantages of the TrEndo with a laparoscopic box trainer compared to a virtual reality (VR) system on a 5-point Likert scale (1 = unimportant, 5 = very important) which we represent as percentages rated low–moderately important (score = 1–3) to highly important (score = 4–5). To assess content validity, participants reflected on the training capacities of the TrEndo within a standardized surgical curriculum using an open questionnaire.

### Construct validity

Participants were asked to correctly position a curved tapered needle into a laparoscopic needle holder (task 1) and complete a standard laparoscopic square knot (task 2) in the laparoscopic box trainer on an artificial skin patch using a 15-cm single 3-0 silk suture. A 5-min time limit was set for task 2. Equipment and instruments used were kept identical. Construct validity was determined by comparison of expert and novice MAPs for both tasks. To explore whether MAPs predict individual laparoscopic skill, a logistic regression analysis was conducted to predict group membership (novice or expert) based on TrEndo performance. Finally, we used a Wald analysis to determine the most contributive MAPs in predicting group membership by calculating individual contributions per MAP to the predictions made in the logistic regression analysis.

### Statistical analysis

Statistical analysis was performed using SPSS 15.0 for Windows (SPSS Inc., Chicago, IL, USA). A nominal significance level of 0.05 was used. All tests were performed two-sided. Values for continuous variables are given as mean (SD), with regard to the multiple TrEndo parameters this was performed using MANOVA analysis, followed by separated ANOVAs. Comparisons with regard to the questionnaire data were performed with two-sided Mann–Whitney *U* analysis. These results are presented as median values. Values for categorical data are specified as frequency (%) and were analyzed using χ^2^ or Fisher’s exact test. A logistic regression analysis was used to test the predictive value of the TrEndo parameters for classification of participants as either novice or expert according to their performance.

## Results

### Demographics, experience, and face validity

The majority of respondents were active in general surgery (Table 1). Thirty-eight experts and 24 trainees returned the questionnaire. Not all respondents completed the entire form, but to calculate mean scores there were never more than four value points missing.

**Table 1:** Participant characteristics

Demographics	Total	Experts	Trainees
Total (male; female)	57 (39; 18)	38 (29; 9)	24 (12; 12)
Mean age (range)		46 (33–60)	32 (28–37)
Respondent specialties	*N*	%	%
General surgery	43	71.1	66.7
Gynecology	9	13.2	16.7
Urology	10	15.8	16.7
Mean no. laparoscopic procedures/year		114.86	40.87
Mean no. complex laparoscopic procedures/year		62.97	7.05

Table 2 depicts mean first impression scores. All questions on first impression were rated above a score of 3 on the 5-point Likert scale. Trainees were slightly more positive than experts, with a significant difference for design and overall functionality.

**Table 2:** Face validity

	Total mean	Experts		Trainees		p*
		Mean	SD	Mean	SD	
Design	3.57	3.36	0.79	3.88	0.74	0.01
Realism	3.30	3.30	0.81	3.29	0.67	0.82
User-friendliness	3.69	3.68	0.66	3.71	0.69	0.81
Overall functionality	3.40	3.08	1.02	3.88	0.74	0.02
Trocar positions	3.79	3.78	0.71	3.79	0.51	0.95
Instrument movement	3.67	3.70	0.85	3.63	0.71	0.65

* Mann–Whitney test, two-sided, expert versus resident

### Content validity

All experts and trainees rated procedural functioning, hand–eye coordination, and depth perception above 3 on the 5-point Likert scale (Table 3). Seventy-five percent of the participants scored 4–5 on the 5-point Likert scale regarding didactic quality compared to a low–moderate score (1–3) by 12.5%. This question was not answered by 12.5% of participants. No significant differences were observed between experts and trainees.

**Table 3:** Content validity

Training capacities	Total mean	Experts		Trainees		p*
		Mean	SD	Mean	SD	
Procedural functioning	4.26	4.16	0.68	4.42	0.56	0.13
Hand–eye coordination	4.31	4.29	0.64	4.35	0.58	0.72
Depth perception	3.48	3.34	0.95	3.69	1.02	0.26

* Mann–Whitney test, two-sided, expert versus trainee

Over 60% of the participants rated each selected advantage of the TrEndo with laparoscopic box trainer as highly advantageous compared to a VR system. No significant difference was observed between experts and trainees (Table 4).

**Table 4:** Selected possible advantages of the box trainer with TrEndo, compared to VR systems

	Total^a^		Experts^a^		Trainees^a^		p*
	1–3	4–5	1–3	4–5	1–3	4–5	
Intro and examples DVD	35.1	64.9	32.4	67.6	39.1	60.9	0.59
Home practice	14.0	86.0	17.6	82.4	8.7	91.3	0.91
Real instruments	5.3	94.7	5.9	94.1	4.3	95.7	0.79
Real needle and thread	0.0	100.0	0.0	100.0	0.0	100.0	1.00
Realistic haptic feedback	13.2	86.8	15.6	84.4	9.5	90.5	0.52
Objective assessment	28.1	71.9	26.5	73.5	30.4	69.6	0.74

Categories are divided into low-to-moderate (1–3) and highly (4–5) advantageous compared to a VR system. All values are expressed as percentages on a 5-point Likert scale

* χ^2^ test, two-sided, expert versus trainees

^a^Valid percent

### Construct validity

Task 1 was completed significantly faster by experts (*n* = 46) than by novices (*n* = 65) (*p* < 0.001). Experts used significantly less path length than novices with both right and left hand and utilized a significantly smaller right-hand area and right-hand volume compared to novices. There were no significant differences between experts and novices with the remaining MAPs, although left-hand area, left-hand volume, and right-hand depth showed a trend in favor of experts.

No novices were able to complete task 2 within 5 min. Table 5 gives the MAPs for task 2, showing a significant difference between novices and experts on all MAPs for both hands.

**Table 5:** Motion analysis parameters for square knot-tying task (task 2)

MAP	Novices		Experts		*p*
	Mean	SD	Mean	SD	
Left path (mm)	6,288.47	1,994.74	2,792.92	1,733.70	<0.01
Left depth (mm)	19.01	5.77	16.19	4.05	<0.01
Left area (deg^2^)	142.53	86.24	89.93	55.13	<0.01
Left volume (mm^3^)	2,746.13	1,790.54	1,318.09	870.48	<0.01
Right path (mm)	5780.33	2096.37	2862.95	1587.96	<0.01
Right depth (mm)	17.46	4.08	14.98	3.62	<0.01
Right area (deg^2^)	104.40	51.52	85.34	40.48	0.03
Right volume (mm^3^)	2,170.82	1,128.60	1,451.15	863.43	<0.01
Time (s)	277.53	39.97	117.59	77.80	<0.01

Based on recorded MAPs it was possible to correctly classify 84.8% of novices (56 of 66) and 84.4% of experts (38 of 45) for task 1. The TrEndo correctly classified 98.3% of novices (58 of 59) and 92.9% of experts (52 of 56) for task 2. Overall, the TrEndo correctly classified 84.7% of participants (94 of 111) at the first task and 95.7% of participants (110 of 115) at the second task.

Left path (Wald score 10.97), left depth (Wald score 4.63), and time (Wald score 15.67) were the most relevant parameters in determining participant level at task 1. At task 2, left volume (Wald score 4.80) and time (Wald score 15.37) were the most contributory parameters to correctly classifying participants as either expert or novice (Table 6).

**Table 6:** Calculated Wald values for needle positioning (task 1) and square knot-tying task (task 2)

Dependent variable	Needle positioning		Square knot	
	Wald	*p*	Wald	*p*
Left path (mm)	10.97	<0.01	0.05	0.82
Left depth (mm)	4.63	0.03	0.09	0.76
Left area (deg^2^)	0.43	0.51	2.38	0.12
Left volume (mm^3^)	1.20	0.27	4.80	0.03
Right path (mm)	0.01	0.92	1.52	0.22
Right depth (mm)	0.06	0.81	0.01	0.92
Right area (deg^2^)	0.27	0.61	0.38	0.54
Right volume (mm^3^)	0.49	0.49	0.60	0.44
Time (s)	15.67	<0.01	15.37	<0.01

## Discussion

Structured objective training and assessment of surgical skills prior to implementation in the OR is a hot topic in current surgical, gynecologic, and urologic education [39]. This is one of the first studies to evaluate laparoscopic suturing tasks using a motion-tracking device on a traditional laparoscopic box trainer. Besides the laparoscopic box trainer, MIS simulators include augmented-reality (AR) and virtual reality (VR) systems [21, 32, 40, 41]. AR simulators combine the physical reality (such as in a box trainer) with virtual reality into one system. VR simulators are completely computer-based; software replicates entire MIS procedures.

AR simulators similar to the TrEndo include the Red Dragon (EDGE) (SimuLab, Seattle, WA, USA) and the ProMis (Haptica Inc., Boston, MA, USA). Both track instrument movements using a passive vision-tracking system, with cameras additionally capturing video images of internal laparoscopic instrument movement. The Red Dragon also provides force measurement on instruments and tissue. These latter two instruments are, however, sensitive to interference and usable only in a laboratory setting [42–44]. Compared to the TrEndo they are also relatively expensive (training option) and are unable to deploy a faculty’s own instruments.

Most AR and VR simulators provide objective feedback after an exercise using incorporated metrics calculated by motion tracking. Such feedback allows supervisors and trainees to monitor performance and progress objectively without supervisor’s presence. Tracking systems are inherently present in VR simulators; however, their use for assessment is often not tested or validated [45]. All MIS simulators aim for a maximum realistic setting but basic laparoscopic skills training in particular remains unrealistic as AR and VR simulators are computer-based. Furthermore, most AR and VR simulators do not provide realistic tactile (haptic) feedback [46]. Previous studies illustrated the importance of haptic feedback during laparoscopic training [47, 48], demonstrating significant improvement of surgical skills in the presence of haptic feedback compared to training in the absence of haptic feedback [32, 48]. VR simulators tend to be expensive and immobile and the advanced technology compromises user-friendliness. Realism and procedural training are limited as VR simulation tends to focus on hand–eye coordination training [49].

Based on recorded MAPs, the TrEndo correctly classified 95.7% of a large and diverse study group into appropriate expert or novice groups. MAPs time, left depth, and left path were most contributory to correctly predicting group membership when positioning a needle into a needle holder, most likely because the left hand is often not used by novices during this task. After the performance of the square knot task, all MAPs differed significantly between experts and novices. Time and left-hand volume were the most contributory MAPs for this task. We believe a difference in novice versus expert dexterity is determined mainly by left-hand skill.

This study follows on the work described by Chmarra et al. [38], in which the TrEndo was able to correctly classify 23 of 31 gynecologists based on laparoscopic nonsuturing tasks, and on the work of Yamaguchi et al. [50], who demonstrated the efficacy of an objective evaluation of psychomotor skills for laparoscopic suturing using an electromagnetic motion-tracking system for MAPs time, left path length, and right speed between experienced surgeons and novice surgeons. In accordance with prior results, this study showed that time and path length help distinguish between expert and novices [22, 51]. We recruited a large and diverse study group, employed an extensive set of MAPs, and focused on laparoscopic tasks, including intracorporeal suturing and knot-tying.

Many novices had difficulty concentrating a full 5 min, and not all recruited experts had equal experience or skill in laparoscopy, and we did not acknowledge a subject’s dominant hand. This may have influenced recorded MAPs.

Metrics currently must still be interpreted by a faculty member. We are investigating facilitation of this interpretation and therefore facilitate experts to novices or intermediate comparison.

Our face and content validity data indicate that the TrEndo would be acceptable to experts and trainees as a training device (including at home). Future studies should investigate the training capacities of the TrEndo. As the TrEndo is user-friendly and mobile it also holds real potential as a home training device. We are currently investigating this autonomous training option.

It is important to realize that MAPs provide an indication of task efficiency and with the exception that no novices completed a knot in this study, knot quality was not evaluated. The comparison of quantitative measures of novice to expert does provide evaluation of novice performance and does confirm construct validity. To provide an integrated objective assessment of MIS skills, efficiency parameters should be combined with other valid metrics of MIS skill. Improved efficiency parameters may correlate with improved quality parameters; however, this should be assessed in future studies. The first step in comparing quality parameters with efficiency indicators may include tasks where it is relatively easy to score task “error” performance (e.g., a stretched surgical glove where subjects must pass a needle through defined targets without tearing). Inclusion of an inexpensive tensiometer might also provide information on quality assessment; however, current tensiometers are too expensive. Besides task quality and efficiency, surgeons also need a core knowledge base, clinical decision-making and communicative skills, and the ability to think and work under stress in a team setting when performing MIS on patients [52, 53]. Future steps should assess the additional value of (recorded TrEndo) efficiency parameters into an integrated assessment of MIS skills and the objective value of (TrEndo-based) efficiency parameters herein.

## Conclusion

Face, content, and construct validities of the TrEndo were established. The TrEndo holds real potential as a (home) training device.
